# A case report: Pain in the hand and tingling of the upper limb may be a symptom of a schwannoma in the supraclavicular region

**DOI:** 10.1016/j.ijscr.2023.108664

**Published:** 2023-08-16

**Authors:** Elena Lanfranchi, Tracy Fairplay, Roberto Tedeschi

**Affiliations:** a Department of Medicine and Health Science “Vincenzo Tiberio”, University of Molise, “Cardarelli Hospital”, 86100 Campobasso, Italy; b Department of Biomedical and Neuromotor Sciences, Alma Mater Studiorum, University of Bologna, Bologna, Italy; c Studio Lanfranchi, Private Practice, Bologna, Italy; d Studio Fairplay - Functional Rehabilitation of the Upper Extremity, Private Practice, Bologna, Italy; e Physical Medicine and Rehabilitation Unit, IRCCS Istituto Ortopedico Rizzoli, 40136 Bologna, Italy

**Keywords:** Brachial plexus, Schwannomas, Hand pain, Physiotherapy, Referral, Tinel

## Abstract

**Introduction:**

Schwannomas, or neurilemmomas, are rare benign nerve sheath tumors primarily originating from peripheral nerves. Brachial plexus schwannomas, constituting approximately 5 % of cases, present a diagnostic and surgical challenge due to their rarity and the complex anatomy of the brachial plexus.

**Case presentation:**

We present the case of a 51-year-old man who visited our physiotherapy clinic with a two-year history of intermittent pain and tingling in the fourth and fifth metacarpals of his non-dominant hand (Numeric Pain Rating Scale 2/10). The pain was nocturnal and resistant to various treatments. Physical examination did not reveal “red flag” symptoms. Considering the persistent and atypical nature of the symptoms, further diagnostic investigations, including an ultrasound of the supraclavicular region, were recommended.

**Clinical discussion:**

Our case report emphasizes the importance of considering brachial plexus schwannomas in patients with prolonged and unconventional symptoms in the fourth and fifth digits, accompanied by supraclavicular swelling and a positive Tinel's sign. Comprehensive diagnostic evaluation is crucial to confirm or rule out a schwannoma in the supraclavicular area. The rarity of such tumors and the intricate brachial plexus anatomy require meticulous diagnostic and surgical approaches.

**Conclusions:**

This case adds to the growing understanding of brachial plexus schwannomas and their diagnostic complexities. Our report underscores the significance of recognizing these tumors in patients with distinct symptomatology and highlights the need for detailed diagnostic assessments and surgical planning.

## Highlights

• Brachial plexus schwannomas are extremely rare tumors
• Man, 51 years old left hand pain, tingling.
• Red flag suspected; referral advised.
• Referral for supraclavicular ultrasound.
• Consider brachial plexus schwannoma in a typical symptoms.

## 1 Introduction

Schwannomas or neurilemmomas are benign nerve sheath tumors composed of Schwann cells of the peripheral nerves [1]. Schwannomas originating from the brachial plexus are rare, accounting for only approximately 5 % of all cases [2], but at the same time when tumors present in this area, Schwannomas and neurofibromas are the most frequently occurring. Most Schwannomas of the brachial plexus are asymptomatic upon diagnosis [2,3], however, they may also be accompanied by secondary symptoms of nerve compression. We present a case that has occurred in our outpatient clinic of a man with intermittently persistent pain and tingling in the 4th and 5th metacarpals of the left hand over the last two years, which was eventually diagnosed as a brachial plexus Schwannoma. We also reviewed similar cases previously reported in the literature. Our case highlights the importance of recognizing that in cases of atypical tingling and pain in the 4th and 5th digit region, especially if the patient presents with a positive Tinel's test in the brachial plexus region, further diagnostic examinations should be conducted and may lead the clinician to rule or rule out the presence of a Schwannoma located in supraclavicular area. Due to the rarity and anatomical complexity of the brachial plexus, Schwannomas in this region represent a diagnostic and surgical challenge [4].

Rashid M et al. [5] also reported two cases of brachial plexus Schwannoma with C7 root involvement that presented with supraclavicular neck swelling [5]. Neurological dysfunction, pain and rapidly growing tumors with suspicion of malignancy are usual indications for surgical resection. Grossly, these tumors are round, oval or plexiform and can be yellow, grey, pink or tan.

Ranjan S et al., emphasizes how an accurate anamnesis and specific evaluation in patients with Schwannoma is decisive for future surgery, and stresses that in more complex cases physiotherapy is important for further functional improvement [4].

## 2 Case presentations

A 51-year-old man presented in our physiotherapy clinic complaining of intermittently persistent pain and tingling in the fourth and fifth metacarpals of his left hand (non-dominant side), (Numeric Pain Rating Scale 2/10) for over the past two years. The pain was also nocturnal, and he could not find any physical posture which reduced his pain. He took anti-inflammatory drugs for a brief period of time, but they were of little to no benefit in reducing his discomfort. He did not have any history of fever, trauma or systemic illness. He had already undergone a cervical MRI (that showed degenerative disc disease of C6-C7) and tried manual physiotherapy of the cervical spine and shoulder girdle in another clinic without resolution of symptoms. On objective examination, active shoulder motion had no restriction, but an increased electric shock sensation along the entire ulnar side of the arm. The Donatelli's tests were performed in order to passively test the capsular tissue of the gleno-humeral joint [6] (Table 1). Only the first position (external rotation at 0° of abduction) was found positive (suggesting a restriction caused by the subscapularis muscle). Since it was observed that active shoulder motion caused an associated electric shock sensation along the arm's ulnar side, additional shoulder motor control tests were performed (Dynamic relocation test and the Modified scapular assistance test). Both of the tests had positive findings.

**Table 1:** Clinical examination.

Shoulder examination:	
Active motion	No restriction of movement, but an increased electric shock sensation was observed along the entire ulnar side of the arm
Passive motion: Donatelli's tests [6] (to test the capsular tissue of the gleno-humeral joints)	- external rotation at 0° of abduction (subscapularis muscle): positive - external rotation at 30° of abduction in the scapular plane (subscapularis muscle): negative; - external rotation at 45° of abduction in the scapular plane (MGHL: anterior capsular tissue): negative; - external rotation at 90° of abduction in the scapular plane (IGHLC - AB: anterior capsular tissue): negative - intra rotation at 30° of abduction in the scapular plane (posterior capsular tissue, inferior part): negative - intra rotation at 30° of abduction and 30° of extension (posterior capsular tissue, superior part): negative - intra rotation at 90° of flexion (posterior capsular tissue): negative
Motor control test: - Dynamic relocation test [11]: positive - Modified scapular assistance test [12]: positive Both tests recreated tremors along the arm's ulnar side	
Palpation	A palpable mass was found in the left clavicular region
Hand assessment	
Global grip strength, (Jamar Hydraulic Hand Dynamometer, position II)	Right hand (dominant side): 13 kg Left hand (affected, non-dominant side): 11 kg Left grip strength = 85 % right grip strength
Pinch strength (Baseline Hydraulic Pinch Gauges in modified tip to tip position including 4th and 5th fingers in opposition to the thumb)	Right hand (dominant side): 4 kg Left hand (affected, non-dominant side): 3 kg Left pinch = 75 % right grip strength
Semmes-Weinstein test [13] for light touch sensibility	Normal
Specific tests for neuropathies - Elbow flexion test [14] (elbow flexed 130°, forearm supinated, wrist extended) between 10 and 60′: negative - Shoulder internal rotation test [15] (Shoulder internal rotation from abducted to 90° and flexed 10°) within 10″: negative - elbow flexion test (patient's hand passively on his 8th rib) within 5″: negative - Scratch collapse test [16]: negative	
Tinel's sign [17]: Negative at Guyon's tunnel and the Cubital tunnel; Highly positive on the medial and upper region of the left clavicle, above the brachial plexus	

During a palpatory examination a palpable mass was found in the left supraclavicular region. The patient's global grip strength, measured by a Jamar Hydraulic Hand Dynamometer in position II, was 13 kg on the right hand (dominant side) and 11 kg the left hand (affected, non-dominant side). The literature is not yet unanimous in defining whether the dominant limb should be stronger than the contralateral side and whether the fact that the patient is right-handed or left-handed affects grip strength [[7], [8], [9]]. In this case, the non-dominant hand grip strength was slightly weaker. Also, the patient's pinch strength was measured by using a Baseline Hydraulic Pinch Gauge. The patient was asked to perform a modified tip to tip position, which postured the 4th and 5th fingers in opposition to the thumb. The strength values obtained in this test also were weaker in the painful-non dominant hand (Table 1). The light touch sensibility test, assessed by the Semmes-Weinstein monofilament test, had normal findings (Table 1).

Lastly, specific tests for upper extremity neuropathies (Elbow flexion test, Shoulder internal rotation test, Shoulder internal rotation test elbow flexion test and Scratch collapse test at the Cubital tunnel), were all performed and presented with negative findings.

Tinel's sign was negative in both the Guyon's tunnel and the Cubital tunnel, but it was highly positive on the medial and upper region of the left clavicle, above the brachial plexus. All the performed tests are presented in Table 1.

**Strangulation Neuropathy Consideration: It is important to note that while not explicitly mentioned, the presence of a positive Tinel's sign above the brachial plexus and the patient's symptoms of pain and tingling in the 4th and 5th metacarpals might indicate the possibility of nerve compression or irritation, potentially resembling strangulation neuropathy. Although this aspect wasn't explicitly addressed in the abstract, the clinical implications of nerve compression were considered in conjunction with the patient's history and examination findings.**

**This case study adheres to the SCARE[****10****](Surgical Case Report) guidelines for reporting surgical case studies. The SCARE guidelines aim to enhance the transparency and completeness of reporting surgical cases, providing a structured framework that facilitates accurate communication and assessment of surgical experiences**.

**Image 1:**
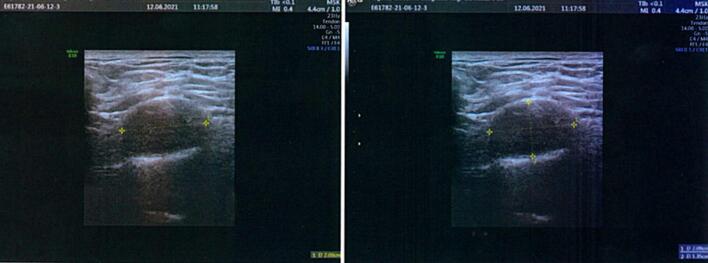
Ecography of the brachial plexus area, showing the presence of a Schwannoma-compatible nodule, suggesting further investigation by MRI.

Based on the clinical examination and the clinical history (the patient's symptoms worsening over time), it was decided to interrupt the physiotherapic examination and to write a letter of referral to his attending physician in order to suggest more specific diagnostic examinations of the brachial plexus region before hypothesizing a motor control program for the shoulder. An ultrasound was conducted and it showed the presence of a hypoechogenic ovalar area of 2 cm × 1.3 cm (Image 1). An MRI and neurosurgical examination for suspected Schwannoma were then suggested. The patient was operated on in May 2022 and to date the symptoms have almost completely disappeared.

## 3 Clinical findings

On physical examination, the patient presented with pain in the fourth and fifth metacarpals of the left hand, intensity 2 out of 10 on the NPRS scale increasing, with a peak score of 8–9 during the last week before evaluation. A highly positive Tinel's sign and a localized swelling in the left clavicle were found. No significant limitation in upper limb range of movement was found, but the shoulder motor control test provoked electric shock sensations along the ulnar aspect of the arm. Global and modified pinch grip were slightly weaker. Sensitive touch sensibility (tested by Semmes-Weinstein monofilament test) in the left hand was not found to be significantly decreased. The patient reported to have occasional back pain when sitting and tinnitus in his left ear. No other comorbidities were reported.

## 4 Diagnostic assessment

### 4.1 Tinel's test

Tinel's test is used to test for compression neuropathy, commonly in diagnosing carpal tunnel syndrome [17].

It is performed by lightly tapping (percussing) over the nerve to elicit a sensation of tingling or “pins and needles” in the distribution of the nerve. The Tinel sign is the tingling or prickling sensation elicited by the percussion of an injured nerve trunk at or distal to the site of the lesion. The sign also indicates nerve regeneration. The test is positive when a tingling or prickling sensation is felt in the distribution of the nerve [17].

Conditions that have been associated with a Positive Tinel's sign [17]:

• Carpal Tunnel syndrome
• Cubital tunnel syndrome
• Radial nerve entrapment
• Tarsal tunnel syndrome
• Superficial peroneal neuropathy, and
• Thoracic outlet syndrome.

## 5 Follow-up and outcomes

After the surgical excision of the Schwannoma, that was performed in May 2022, the pain and tingling in the patient's upper limb gradually stopped. At the present time a follow up examination has not yet been conducted.

## 6 Discussion

This article points out the important role of a thorough multidisiciplinary diagnosis in this particular case where the patient, for numerous years, has presented with pain in his 4th and 5th metacarpals and a painless swelling in the left supraclavicular region. His symptom presentation can easily be misdiagnosed in a clinical practice as an enlarged lymph node, or, in our specific case, were the motor control tests for the shoulder were positive, as a shoulder motor control dysfunction. As in our clinical case, it was only possible to make the rare diagnosis of a brachial plexus Schwannoma by combining the clinical examination expertise of the physiotherapist with specific instrumental diagnostic tests.

In conclusion, Schwannomas of the brachial plexus should be considered as a differential diagnosis in patients who present with long-standing 4th and 5th metacarpal pain and tingling, combined with supraclavicular swelling. Since these are potentially treatable lesions, it can be strongly suggested that a detailed history and diagnostic examinations are performed in association with a medical referral before starting physiotherapy treatment sessions. In these ambiguous cases, as has been verified in our case report, it is probable that the physician will prescribe ultrasound and imaging studies (MRI) in order to assess the supraclavicular region first and to verify if a nodule is present and thus determine its entity/Schwannoma. In this manner a pre-operative diagnosis can be made, thus reducing the chances of clinical mismanagement. Before starting a physiotherapy protocol for motor control of the shoulder, various “red flag” symptoms had to be excluded. It is for this reason we wrote a referral letter to the attending physician in order to report the supraclavicular swelling, positive Tinel's sign and palpable nodule in the brachial plexus area. When in doubt and “red flag” symptoms are present upon physiotherapy clinical evaluation, the physiotherapist should alert the physician with a written referral in order to report all the patient's symptoms, clinical test findings and to ask for further diagnostic investigation before starting the physical therapy treatment. This way of approaching our patients' treatment, not only ensures the most appropriate management and care of our patients but also upholds the integrity and professional responsibility of our profession. The CARE Checklist was followed for the writing of this article [18].

## Ethical approval

Our institution does not require ethical approval for reporting individual cases or case series.

## Funding

Authors state no funding involved.

## Author contribution

RT and EL contributed to conception and design of the study; RT to data acquisition, RT and EL to data analysis and interpretation; RT and EL and TF contributed to draft the manuscript; RT and EL contributed to the critical revision for important intellectual content. All authors read and approved the final version of the manuscript.

## Guarantor

Roberto Tedeschi

## Research registration number

N/A

## Informed consent

Informed consent has been obtained from all individuals included in this study.

## Conflict of interest statement

Authors state no conflict of interest.

## Data Availability

The protocol and the dataset analyzed during the current study is available from the corresponding author on reasonable request.

## References

[1] Dai A., Cai J.-P. (2021). Intravascular schwannoma: a review of a rare diagnosis. J. Cutan. Pathol..

[2] Kumar A., Akhtar S. (2011). Schwannoma of brachial plexus. Indian J. Surg..

[3] Chen F., Miyahara R., Matsunaga Y., Koyama T. (2008). Schwannoma of the brachial plexus presenting as an enlarging cystic mass: report of a case. Ann. Thorac. Cardiovasc. Surg..

[4] Ranjan S., Arora N., Sethi D., Kaur D., Sethi G. (2020). Schwannoma of the brachial plexus: a rare case report. Iran. J. Otorhinolaryngol..

[5] R. M, S. O, Y. S, Q. Ua, Y. K (2013). Schwannoma of the brachial plexus; report of two cases involving the C7 root. J. Brachial Plex. Peripher. Nerve Inj..

[6] Donatelli R. (2012).

[7] El-Gohary T.M., Abd Elkader S.M., Al-Shenqiti A.M., Ibrahim M.I. (2019). Assessment of hand-grip and key-pinch strength at three arm positions among healthy college students: dominant versus non-dominant hand. J. Taibah Univ. Med. Sci..

[8] Ertem K., Inan M., Yoloğlu S., Elmalı N., Hanna A., Sabina S., Bora A. (2003). Effects of dominance, _body mass index and age on grip and pinch strengt11. Isokinet. Exerc. Sci..

[9] Schmidt R.T., Toews J.V. (1970). Grip strength as measured by the Jamar dynamometer. Arch. Phys. Med. Rehabil..

[10] Agha R.A., Franchi T., Sohrabi C., Mathew G., Kerwan A., SCARE Group (2020). The SCARE 2020 Guideline: updating consensus Surgical CAse REport (SCARE) guidelines. Int. J. Surg..

[11] Magarey M., Jones M. (2003). Specific evaluation of the function of force couples relevant for stabilization of the glenohumeral joint. Man. Ther..

[12] Seitz A.L., McClure P.W., Lynch S.S., Ketchum J.M., Michener L.A. (2012). Effects of scapular dyskinesis and scapular assistance test on subacromial space during static arm elevation. J. Shoulder Elb. Surg..

[13] Yildirim P., Gunduz O.H. (2015). What is the role of Semmes-Weinstein monofilament testing in the diagnosis of electrophysiologically graded carpal tunnel syndrome?. J. Phys. Ther. Sci..

[14] Rosati M., Martignoni R., Spagnolli G., Nesti C., Lisanti M. (1998). Clinical validity of the elbow flexion test for the diagnosis of ulnar nerve compression at the cubital tunnel. Acta Orthop. Belg..

[15] Ochi K., Horiuchi Y., Tanabe A., Waseda M., Kaneko Y., Koyanagi T. (2012). Shoulder internal rotation elbow flexion test for diagnosing cubital tunnel syndrome. J. Shoulder Elb. Surg..

[16] Čebron U., Curtin C.M. (2018). The scratch collapse test: a systematic review. J. Plast. Reconstr. Aesthet. Surg..

[17] Moldaver J. (1978). Tinel’s sign. Its characteristics and significance. J. Bone Joint Surg. Am..

[18] CARE Checklist, CARE Case Report Guidelines. https://www.care-statement.org/checklist.

